# Practical Remarks on Gout, Rheumatic Fever, and Chronic Rheumatism of the Joints, &c.

**Published:** 1843-08-05

**Authors:** 


*Practical Remarks on Gout, Rheumatic Fever, and Chronic Rheumatism of the Joints, &c.*

BY DR. TODD.

The sebaceous glands are not so numerous [as the sudoriferous ones;] they are most abundant in the vicinity of hairs. Their form is that of smal vesicular bags, which open by minute orifices into l hair follicle, or quite close to one. When sebace oa matter is suffered to accumulate in these glands, a peculiar disease of the skin is induced, called *acne*, which often shows itself on the face, nose, or forehead, and very frequently on the back. In a simple form the accumulations are denoted by numerous black points, produced by particles of dust being entangled in the sebaceous matter, which chokes the orifices of the glands. The skin around them will often inflame, and angry pustules result.

Nothing favours the excretion of this sebaceous matter so much as cleanliness and friction. If any arguments were wanting to enforce the propriety of adopting means for these purposes, it is derived from the curious, and in some measure humiliating, fact lately discovered by Dr. Simon of Berlin, that these glands are the habitat of a parasitic insect, which has been called the *entozoon folliculorum.* This creature is of considerable size, and may exist alone, or in clusters of several, in a single gland. In the perfectly healthy state they are few in number; but when sebaceous matter, their proper food, is suffered to accumulate, they abound. Through the kindness of my friend, Mr. Erasmus Wilson, who has lately read a paper to the Royal Society on their structure and habits, I have been enabled to see the insect alive and had a favourable opportunity of watching its movements, as well as carefully observing its form and structure. Cleanliness and friction remove sebaceous matter, and, therefore, oppose the accumulation of those insects; and the local application of a solution of corrosive sublimate is often very beneficial in removing the points of *acne*, which result from the retention of the sebaceous secretion."—*Lond. Med. Gaz.*

